# Transcriptome analysis of the curry tree (*Bergera koenigii* L., Rutaceae) during leaf development

**DOI:** 10.1038/s41598-019-40227-z

**Published:** 2019-03-12

**Authors:** Vikram S. Shivakumar, Gabriel Johnson, Elizabeth A. Zimmer

**Affiliations:** 1 Department of Botany and Laboratories of Analytical Biology, National Museum of Natural History, Smithsonian Institution, PO Box 37012, Washington, DC 20013-7012 USA; 2 Thomas Jefferson High School for Science and Technology, 6560 Braddock Rd, Alexandria, VA 22312 USA

## Abstract

The curry tree (*Bergera koenigii* L.) is a widely cultivated plant used in South Asian cooking. Next-generation sequencing was used to generate the transcriptome of the curry leaf to detect changes in gene expression during leaf development, such as those genes involved in the production of oils which lend the leaf its characteristic taste, aroma, and medicinal properties. Using abundance estimation (RSEM) and differential expression analysis, genes that were significantly differentially expressed were identified. The transcriptome was annotated with BLASTx using the non-redundant (nr) protein database, and Gene Ontology (GO) terms were assigned based on the top BLAST hit using Blast2GO. Lastly, functional enrichment of the assigned GO terms was analyzed for genes that were significantly differentially expressed. Of the most enriched GO categories, pathways involved in cell wall, membrane, and lignin synthesis were found to be most upregulated in immature leaf tissue, possibly due to the growth and expansion of the leaf tissue. Terpene synthases, which synthesize monoterpenes and sesquiterpenes, which comprise much of the curry essential oil, were found to be significantly upregulated in mature leaf tissue, suggesting that oil production increases later in leaf development. Enzymes involved in pigment production were also significantly upregulated in mature leaves. The findings were based on computational estimates of gene expression from RNA-seq data, and further study is warranted to validate these results using targeted techniques, such as quantitative PCR.

## Introduction

The Rutaceae, a family of angiosperms within the order Sapindales, contains around 1730 species among 158 genera^1^ Morton and Telmer (2014) characterized the family into four subfamilies, the Amyridoideae, Aurantioideae, Cneoroideae, and Rutoideae^2^. Among the species in the Aurantioideae subfamily (also known as the Citrus subfamily), the most widely known and cultivated species in the family are those of the genus *Citrus*, most notably the orange (*C*. x *sinensis* (L.) Osbeck), lemon (*C*. x *limon* (L.) Osbeck), and key lime (*C*. x *aurantiifolia* (Christm.) Swingle)^3^.

Within the same subfamily is the tribe Clauseneae, of which the most notable species is *Bergera koenigii* L. (formerly *Murraya koenigii* L.), commonly known as the curry tree. Cultivated for its aromatic leaves, the curry tree is natively grown in tropical and subtropical climates and commonly found in South Asian cooking as a spice, as well as used in traditional Ayurvedic medicine^4,5^.

Recent studies have characterized the medicinal value of the curry plant, finding the leaves to have anti-diabetic^5–7^, anti-oxidative^8,9^, anti-inflammatory^10^, and anti-cancer effects^11,12^. Many of the medicinal properties are attributed to the oil composition of the leaves, which yields the pungent, bitter taste, and aroma favored in South Asian cooking.

The oil composition of the curry leaf has been well characterized in previous studies. Using GC (Gas chromatography) and GC/MS (Gas chromatography/mass spectrometry) with trichlorofluoromethane as a solvent, MacLeod and Pieris (1982) found four main constituents of the curry essential oil: β-phellandrene, caryophyllene, β-gurjunene, and β-elemene^13^. Wong and Tie (1993) used steam distillation along with GC and GC/MS and found caryophyllene and β-phellandrene as well, along with α-pinene^14^. In another study, Mallavarapu *et al*. (1999) found two major oils, α-pinene and sabinene^15^. Lastly, Chowdhury *et al*. (2008) found Caryophyllene and 3-Carene to be major constituents as well^16^.

Caryophyllene, in particular, is a main constituent of the essential oils of both *Piper nigrum* (black pepper) and *Eugenia caryophyllata* (clove), yielding a similar spice to the taste of these three phylogenetically unrelated plants^17,18^. Studies have also found anti-inflammatory properties in the oil^19^, similar to the properties found in curry leaves by Darvekar *et al*.^10^ Verma *et al*. (2012) found that the distinct aroma profile of the curry tree consists of Pinene, Limonene, Caryophyllene, and other monoterpenes^20^. β-phellandrene, a common oil found in various *Citrus* species^21^, as well as in the essential oil of *Eucalyptus* species^22^, may also contribute to this aroma along with Pinenes, which are notable for their pine aroma^23^.

The majority of the essential oils of the curry leaf are classified as terpenes, which act mainly to defend against insects and microbes, as well as to attract pollinators^24^. Aside from the monoterpene and sesquiterpenes found in the essential oil of the curry leaf, another terpene commonly found in plants is gibberellin^24^, which has been found to hormonally regulate leaf development and senescence in various plant organs and tissues^25^.

In this study, the transcriptome of the curry leaf was sequenced to understand the changes in mRNA expression during development to discover the genetic regulation of leaf growth and the pathways that are involved in essential oil production. Due to its widespread cultivation, as well as its medicinal and culinary use, further study into the leaf development of this species will provide valuable data for horticultural applications as well as further elucidate the basic molecular regulation of leaf cell differentiation.

We hypothesize that increased expression will be most apparent among leaf ontogeny genes as the leaf matures, as well as metabolic pathway genes expressed during leaf expansion and cell maturation. We also propose that there would be an increase in the expression of genes involved in the anabolism of secondary metabolites, oils, and pigments as the leaf grows. An increase in pigment producing enzymes is also expected as the larger mature leaves require more chlorophyll for photosynthesis necessary to sustain a greater tissue size^26^.

## Materials and Methods

### Tissue Collection

**Figure 1:**
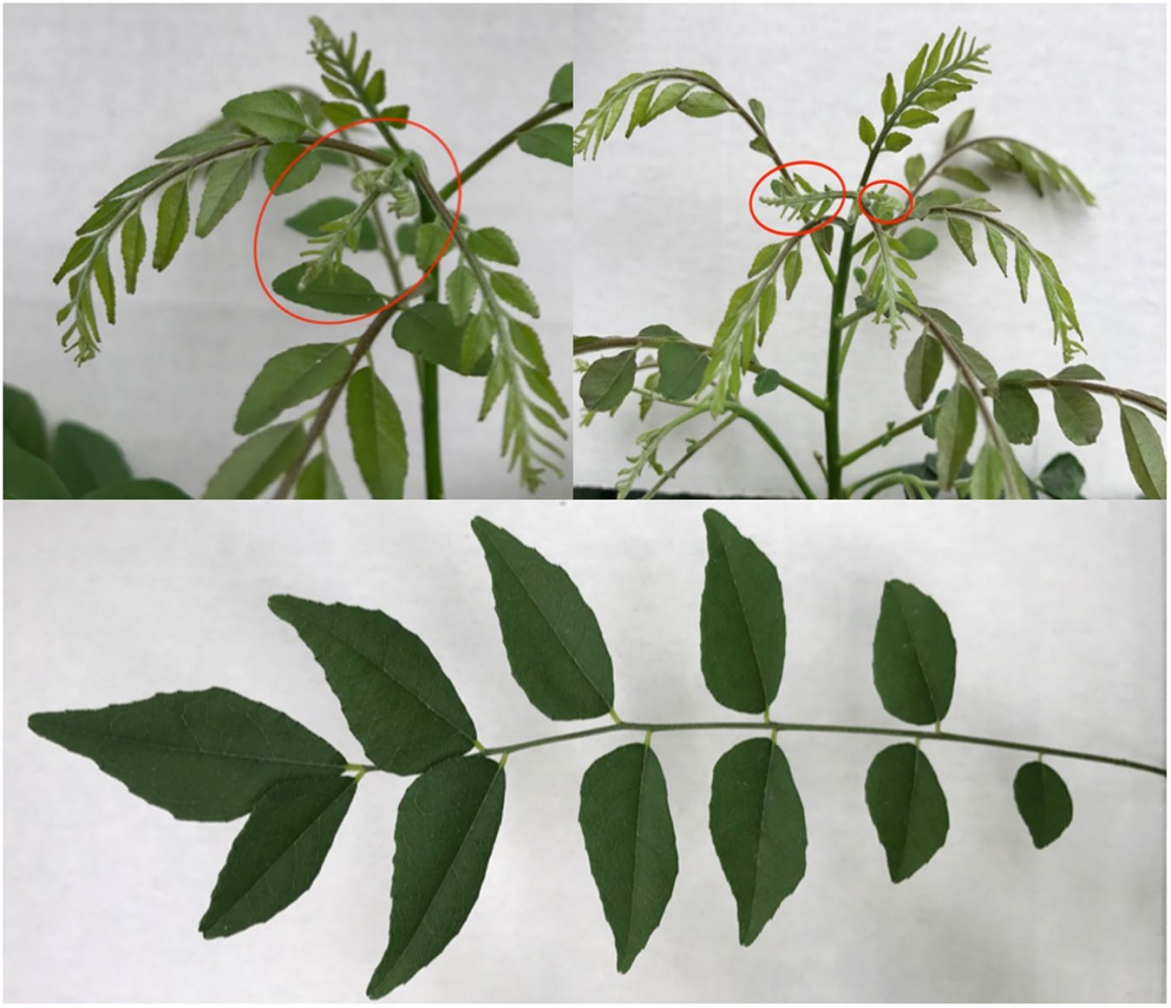
From top-left clockwise: (1) A cluster of immature leaves circled in red, (2) Immature leaves in a cluster at the top of the tree stem, (3) A fully mature leaf.

Both immature and mature leaf tissue (differentiated by size and proximity to the apical meristem, with immature leaves ranging from 0.5–1.0 cm, and mature leaflets from 1.5–3.0 cm) were collected from the curry tree growing in the United States Botanic Gardens in Washington, D.C. (accession 06–0532). The voucher for this *Bergera koenigii* L. specimen is Van Neste 229 (US). Leaf samples were immediately stored in liquid nitrogen at −190 °C. Figure 1 shows both immature and mature leaves from the tree used in this study, located in the US Botanic Garden.

### RNA Extraction and Library Preparation

RNA was extracted from ~50 mg leaf tissue using the Spectrum™ Plant Total RNA Kit (Sigma-Aldrich, St. Louis, USA), following a modified procedure detailed in the kit manual designed for highly recalcitrant *Citrus* tissue, rich in secondary metabolites and oils. Prior to the Spectrum™ Plant Total RNA Kit, various RNA extraction protocols were used on the curry tissue with poor results. The ISOLATE II RNA Mini Kit (Bioline, Taunton, MA, USA), Trizol RNA extraction^27^, and a guanidinium-free phenol extraction designed for use on tissues rich in secondary metabolites^28^, all yielded RNA extracts of low quality and concentration.

The concentrations of the extracted RNAs were determined using a NanoDrop 1000 Spectrophotometer (Thermo Fisher Scientific, Waltham, MA, USA). Extractions with concentrations greater than 25 ng/μL and a 260/280 absorbance ratio of 2.0 ± 0.1 were used for cDNA library preparation. The RNA extractions were sent to the Transcriptome and Genome Analysis Laboratory at the University of Göttingen (Göttingen, Germany) for library preparation and sequencing. There, RNA quality measured by RIN (RNA Integrity Number) was quantified using the Agilent 2100 Bioanalyzer (Agilent Technologies, Palo Alto, CA, USA) before the sequence library was prepared using the TruSeq RNA Sample Preparation Kit (Illumina Inc., San Diego, CA, USA). This library was quantified using the QuantiFluor dsDNA System (Promega, Madison, WI, USA), and size selected using the Bioanalyzer 2100 (Agilent, Santa Clara, CA, USA). Next-generation sequencing (NGS) was performed on the Illumina HiSeq. 4000 instrument (Illumina Inc., San Diego, CA, USA) following standard protocols.

### Data Analyses

Around 76 million ~50 bp single-end sequencing reads were filtered by quality score using the FASTX-Toolkit (http://hannonlab.cshl.edu/fastx_toolkit) and assembled with Trinity 2.1.1^29^ with default parameters. To determine the completeness of the assembly, 2121 single-copy orthologs standard to eudicots were aligned using Benchmarking Universal Single-Copy Orthologs (BUSCO) to the transcriptome^30^. The completeness of the assembly was compared with that of a previously published transcriptome of the curry leaf^31^. The assembled transcriptome was annotated using BLASTx^32^, which searches translated nucleotide sequences against the non-redundant (nr) BLAST database. Sequence descriptions for assembled transcripts were transferred from homologous BLAST hits with E-values <10^−4^. BLAST hits were then functionally annotated using Blast2GO^33,34^ and Gene Ontology (GO) terms were assigned to transcripts with BLAST hits to understand gene function in the leaf tissue^35^.

The Trinity toolkit^36^ was used to facilitate downstream analysis, including abundance estimation, differential expression, and GO enrichment. RNA-Seq by Expectation-Maximization (RSEM) was used for alignment-based abundance estimation^37^ using Bowtie2^38^. Since the assembled transcriptome represents all the transcripts found in the leaf in both stages, the sequence reads from each sample were aligned to the assembled transcriptome to differentiate transcripts unique to one stage or present throughout leaf development. The read counts for each transcript were analyzed using edgeR^39^, a Bioconductor package^40^ used for differential expression analysis, and the logarithmic fold change and false detection rate (FDR) were calculated for each transcript. The results of differential expression and GO annotation were combined for Gene Ontology enrichment using GOseq^41^, which identifies GO terms enriched as a result of differential expression between samples.

## Results

### Transcriptome Assembly Statistics

**Figure 2:**
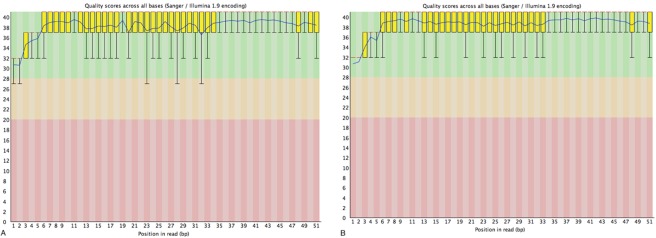
Average quality scores per base in single-end 50 bp sequencing reads from Illumina HiSeq 4000 sequence libraries; (**A**) Immature; (**B**) Mature.

**Figure 3:**
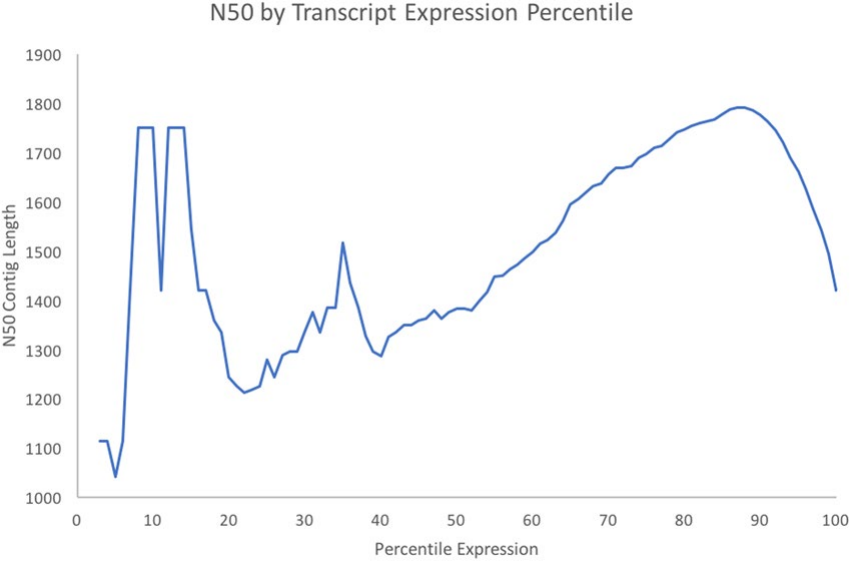
The N50 statistic computed using subsets of highly expressed genes. Percentile expression represents the minimum percentile of expression for which to include a gene in the N50 calculation. The N50 value represents the contig length at which half the assembled bases in the assembly can be found. The peak N50 contig length is reached at the 90th percentile, indicating a high-quality transcriptome assembly.

A transcriptome for the curry leaf was assembled using ~76 million single-end sequence reads, pooled from both immature and mature leaf libraries such that the final assembly shows a more accurate estimation of genes expressed throughout leaf ontogeny. Figure 2 shows the quality scores for each library after quality filtering of the reads. In the leaf transcriptome, 55,441 genes were assembled, comprising of 57,428 transcripts, with a median contig length of 362 bp. The N50 statistic for the transcriptome assembly was 1421 bp, representing the minimum contig length at which half the assembled bases can be found. Although the N50 statistic is similar to the median contig length, it inherently gives a larger weighting to longer contigs, resulting in a longer length compared to the median contig. Figure 3 shows a plot of the expressed N50 (exN50) value, which represents the N50 value computed using only a subset of genes that are highly expressed at a given expression percentile. The leaf transcriptome assembly reaches a peak exN50 at the 90^th^ percentile expression, indicating a high-quality assembly and adequate sequencing depth.

Benchmarking Universal Single-Copy Orthologs (BUSCO) was used to assess the completeness of the transcriptome assembly. By searching for 2121 single-copy orthologs standard in eudicots, the assembly quality of the transcriptome can be quantitatively determined. Of the 2121 benchmark single-copy orthologs, 1726 (83.1%) were complete and present in the assembly (of which 1520 [71.1%] were single-copy and 242 [11.4%] were found to be duplicated), 217 (10.2%) were present but fragmented, and 142 (6.7%) were missing from the assembly altogether. Less than 10% of orthologs were missing from the assembly, indicating a complete and high-quality assembly.

**Figure 4:**
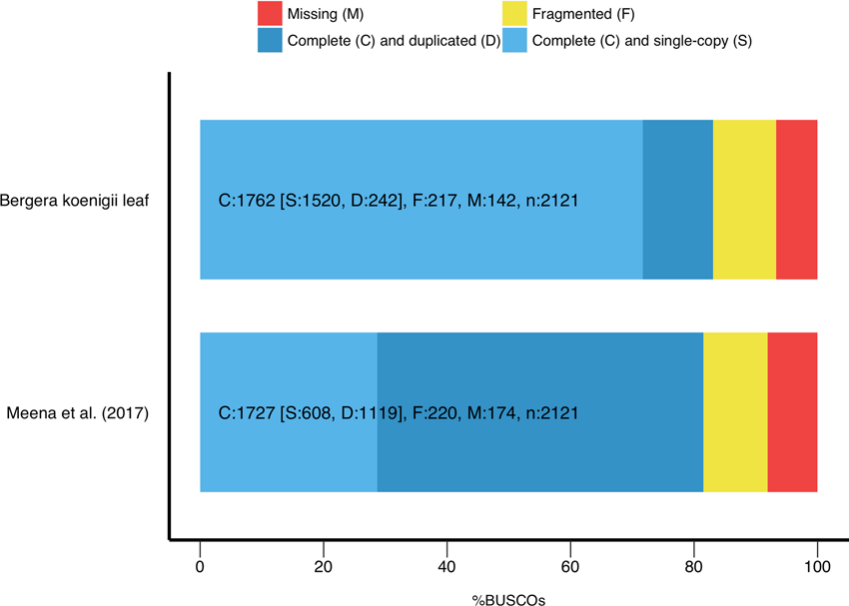
A comparison of the completeness of the newly sequenced transcriptome to that of a previously published curry leaf transcriptome (Meena *et al*.^31^). Using Benchmarking Universal Single-Copy Orthologs (BUSCO), 2121 single-copy orthologs standard to eudicots were searched against each transcriptome to determine if they were found complete (single-copy/duplicated), fragmented, or missing. The newly-assembled transcriptome was found to be more complete than the previously published transcriptome, and fewer duplications of the single-copy benchmark orthologs were found.

BUSCO was run on a previously published transcriptome for the curry leaf^31^, and the results were compared. Figure 4 shows the comparison of the completeness analysis between the two transcriptomes. This transcriptome was found to be more complete than the previously published curry leaf transcriptome, including more complete and fragmented benchmark single-copy orthologs. Of the complete orthologs present in both transcriptomes, the newly assembled transcriptome contained fewer duplications, indicating a higher quality assembly (since the benchmark orthologs used in the analysis were single-copy in origin).

### Transcript Abundance

Using alignment-based abundance estimation, the Fragment Per Kilobase of transcript per Million mapped reads (FPKM) value was calculated for each gene, and a threshold of 1 FPKM was used to determine if a gene was expressed in the sample. Of the 55,441 genes, 13,221 genes were unique to mature leaf tissue, 2449 were unique to immature leaf tissue, 37,271 were common to both developmental stages, and 2,500 genes were found to not be significantly expressed in either sample (FPKM values below the threshold).

### Differential Expression

**Figure 5:**
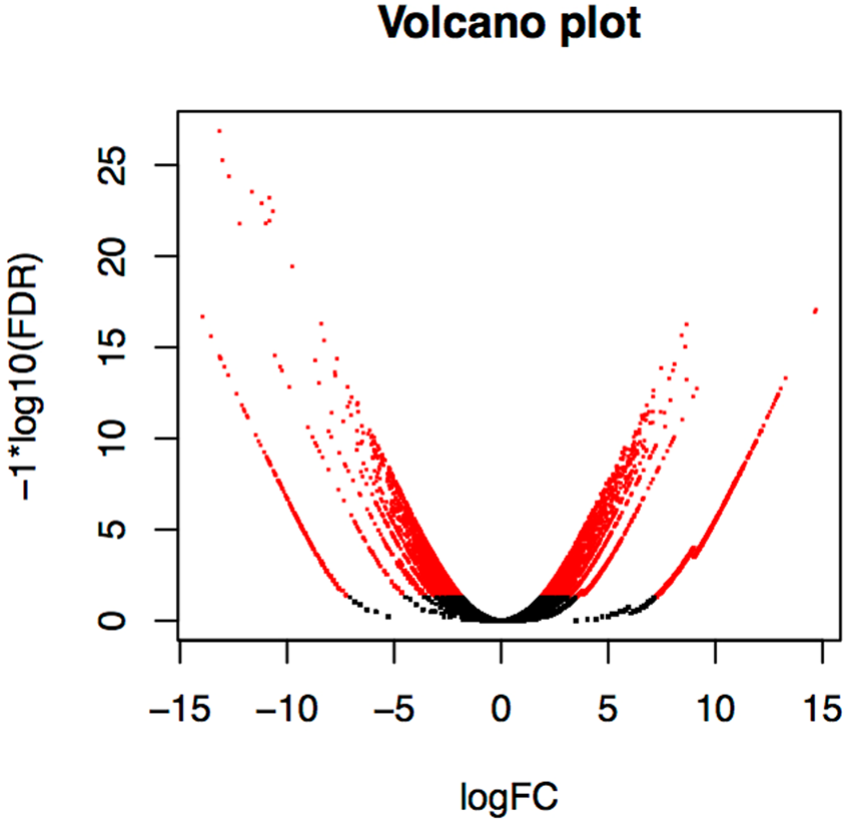
Volcano plot showing the spread of genes based on fold change and False Discovery Rate (FDR), representing statistical significance. Genes with an FDR significance <0.05 are labelled in red.

**Figure 6:**
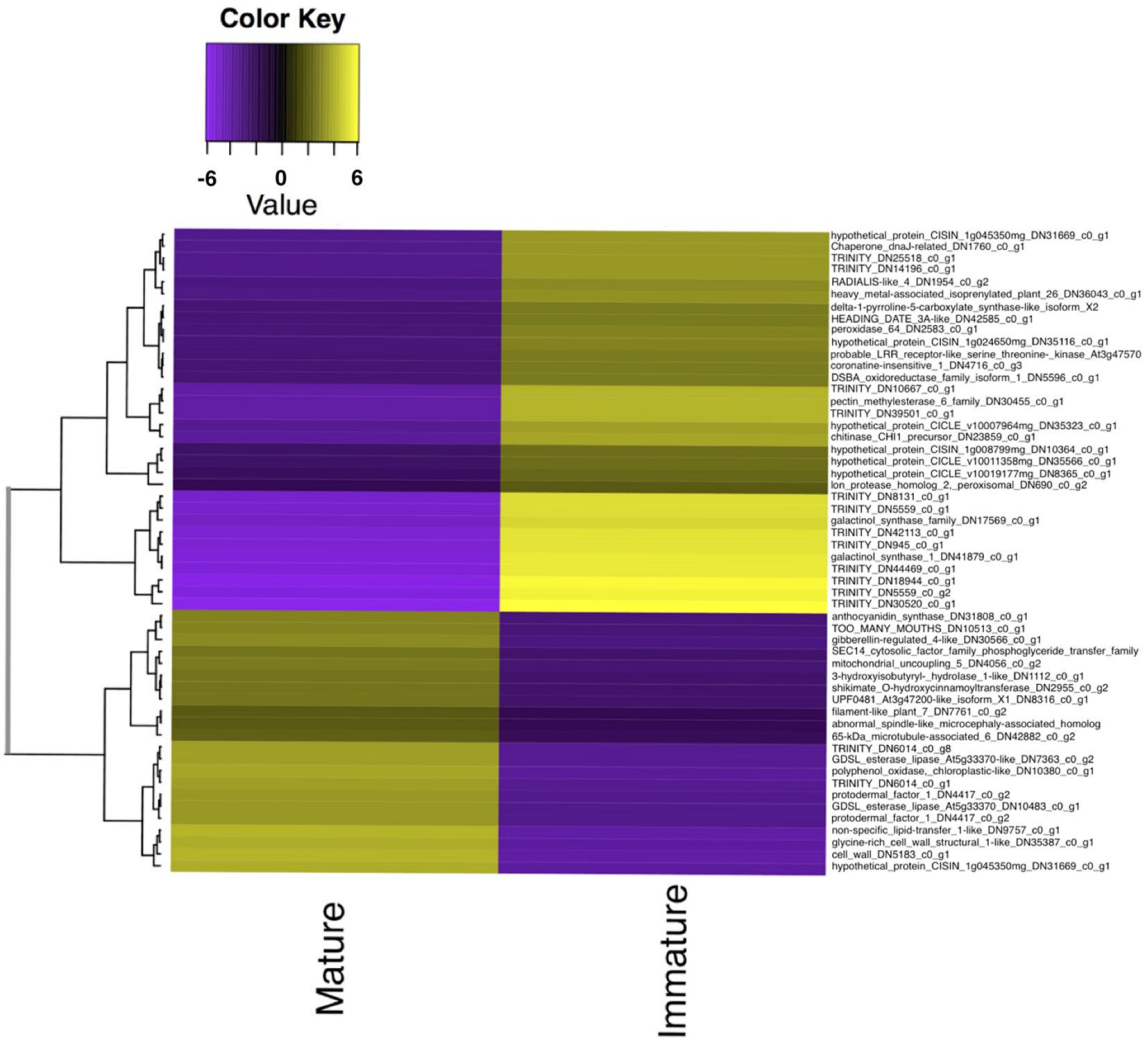
A heat map showing the expression patterns of significantly differentially expressed genes, defined as having greater than 6-fold (a factor of 64) change in expression and FDR <10^−12^, between immature and mature leaf tissue. Genes without a blast hit are listed with a unique transcript ID assigned at assembly. Coloring of rows represents the fold change [log2(exprA/exprB)] calculated from the Fragment Per Kilobase of transcript per Million mapped reads (FPKM) value.

Abundance estimations were compared across samples to calculate the fold change for each gene, along with the false detection rate (FDR), that represents the statistical significance of the differential expression test. Figure 5 shows each gene plotted by its fold change and FDR, displaying genes with an FDR <0.05 in red. For this study, genes expressed with a greater than 6-fold (a factor of 64) change and an FDR <10^−12^ between the two developmental stages were considered significantly differentially expressed. Of the 55,441 genes in the transcriptome, 54 genes were found to be significantly differentially expressed between the two samples. Figure 6 shows the significantly differentially expressed genes, clustered by expression pattern, in a heatmap.

### Annotation and GO Enrichment

**Figure 7:**
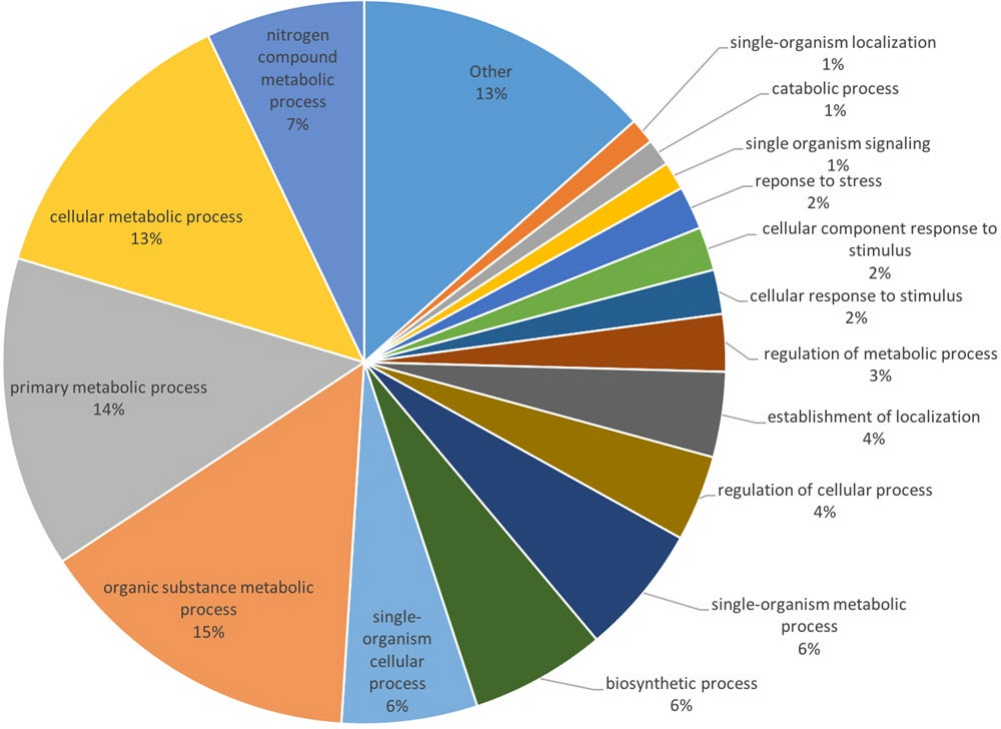
The distribution of functional gene group expression in the leaf transcriptome of *Bergera koenigii*. Categories were based on GO terms assigned after functional annotation.

Transcripts were annotated using BLASTx, assigning a sequence description using the top five BLAST hits with E-values <10^−4^. Through BLASTx, 33,427 transcripts (58.2% of the transcriptome) were annotated with a description based on sequence homology. The remaining ~40% may not have yielded any BLAST hits due to the lack of sequence data available for this species, with the closest well-annotated species being *Citrus sinensis*. These transcripts were functionally annotated using Gene Ontology (GO) terms to understand the role of the gene in the tissue. Of the 33,427 transcripts annotated using BLASTx, 23,650 (70.8%) were assigned GO terms. Figure 7 shows the spread and representation of GO categories expressed in the leaf transcriptome. Combining the results of GO annotation with the differential expression analysis, GO terms which were significantly enriched or depleted as a result of differentially expressed transcripts were identified. In immature tissue, 43 GO terms were found to be enriched due to the upregulation of genes in the sample, and 30 GO terms were found to be enriched in mature tissue.

**Figure 8:**
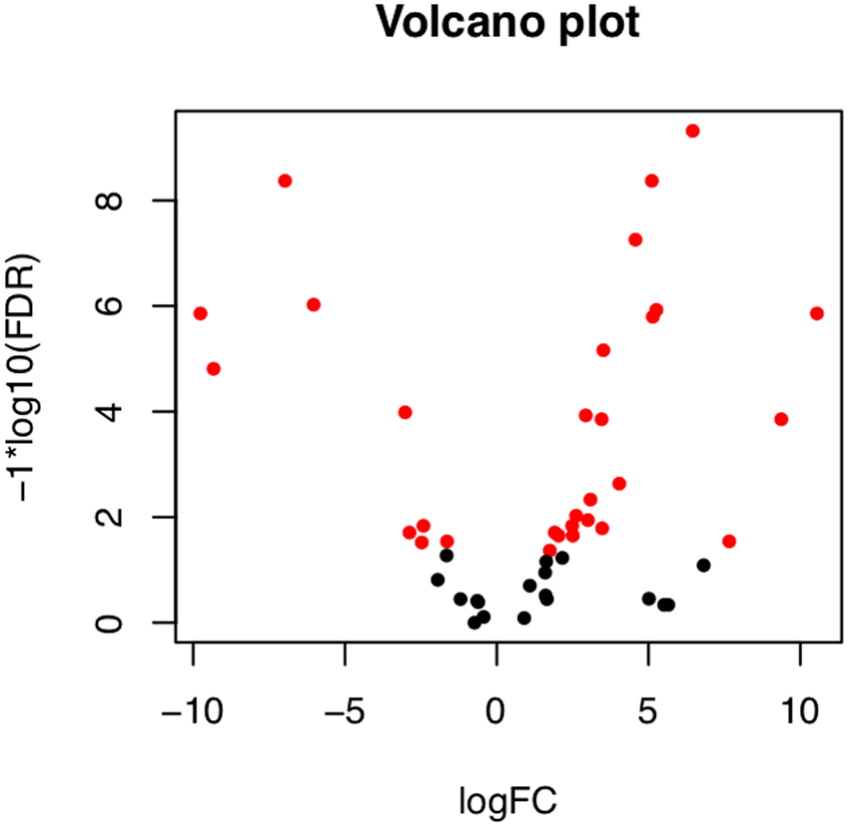
Volcano plot showing the spread of terpene synthase genes based on fold change and False Discovery Rate (FDR), representing statistical significance. Genes with an FDR significance <0.05 are labelled in red.

**Figure 9:**
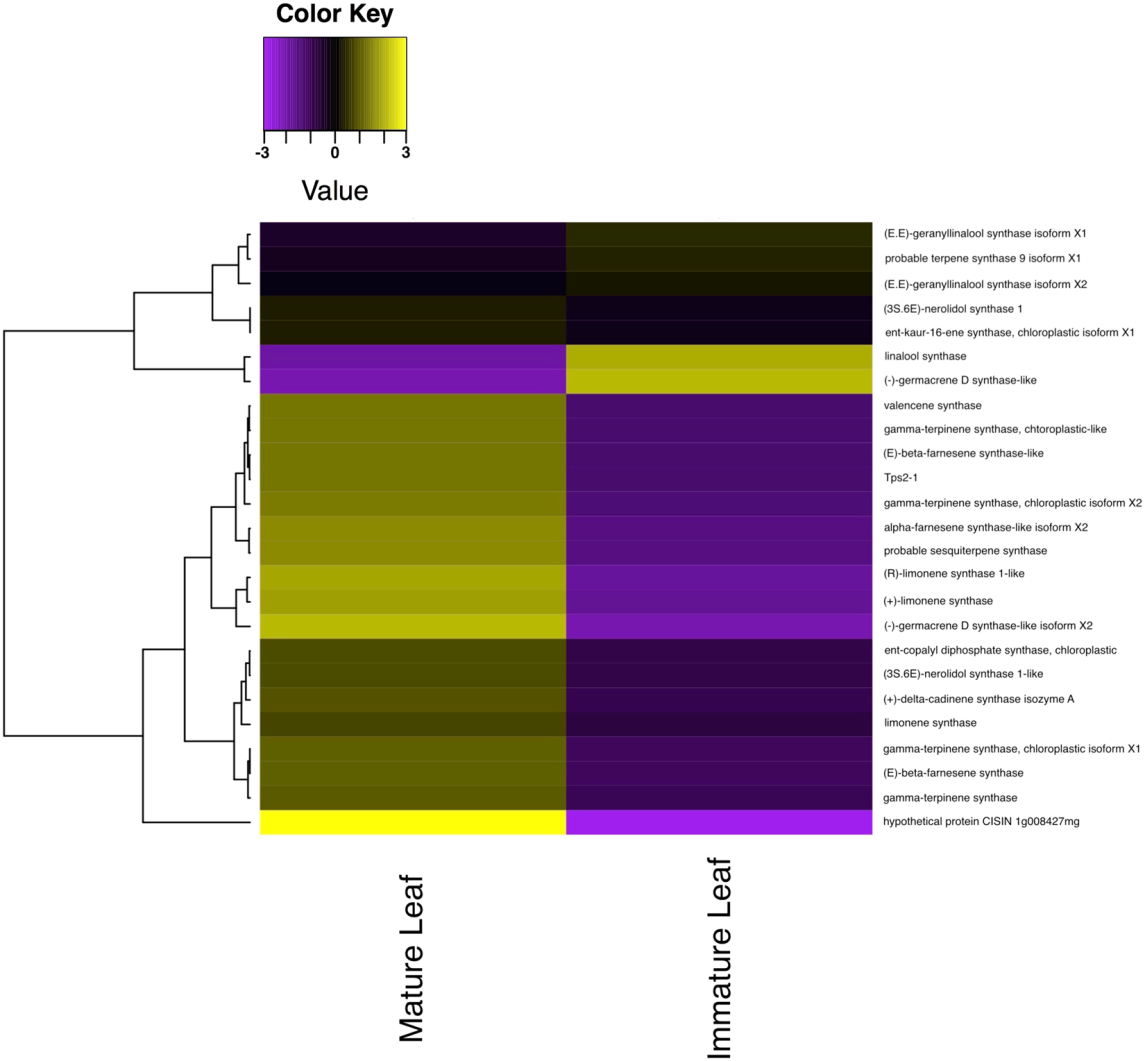
A heat map showing 25 transcripts involved in the terpene biosynthetic pathway that are most differentially expressed, identified by the GO term GO:0010333. All 25 transcripts have FDR values <0.05. Transcripts are labelled with the name of the closest homologous match from BLAST. Coloring of rows represents the fold change [log2(exprA/exprB)] calculated from the Fragment Per Kilobase of transcript per Million mapped reads (FPKM) value.

After assigning GO terms, transcripts involved in terpene biosynthesis were identified by searching the assigned GO terms. 48 genes were identified with the GO term “GO:0010333”, which represents terpene synthase activity. The differential expression of the identified terpene synthase transcripts was studied to compare terpene biosynthetic activity between the leaf developmental stages. Figure 8, similar to Fig. 5, shows each terpene synthase gene plotted by its fold change and FDR, displaying genes with an FDR <0.05 in red. Figure 9 shows the 25 most differentially expressed terpene synthase genes, labelled with the name of the closest homologous match from each respective BLAST query.

## Discussion

### GO Enrichment in Immature Tissue

GO enrichment analysis reveals information about which pathways likely were upregulated as a result of differential expression between the two developmental stages. In the immature leaf tissue, 13 out of 43 enriched GO terms were involved in the building or destruction of cell walls and other structural features of the tissue, such as the lignification of vascular tissue. Table 1 lists the GO terms related to structural activity in the immature leaf along with statistical significance.

**Table 1:** Structural development GO terms significantly enriched in immature leaf tissue as a result of differential expression.

GO ID	*p*-value	# Genes Upregulated	GO Term	GO Category
GO:0046274	7.042E-04	2	lignin catabolic process	Biological Process
GO:0052716	7.042E-04	2	hydroquinone: oxygen oxidoreductase activity	Molecular Function
GO:0005576	1.303E-03	3	extracellular region	Cellular Component
GO:0042744	1.892E-03	2	hydrogen peroxide catabolic process	Biological Process
GO:0042545	2.637E-03	2	cell wall modification	Biological Process
GO:0045330	2.637E-03	2	aspartyl esterase activity	Molecular Function
GO:0030599	2.732E-03	2	pectinesterase activity	Molecular Function
GO:0004601	5.019E-03	2	peroxidase activity	Molecular Function
GO:0045490	7.386E-03	2	pectin catabolic process	Biological Process
GO:0046910	1.191E-02	1	pectinesterase inhibitor activity	Molecular Function
GO:0009809	1.528E-02	1	lignin biosynthetic process	Biological Process
GO:0016998	1.785E-02	1	cell wall macromolecule catabolic process	Biological Process
GO:0005618	1.892E-02	2	cell wall	Cellular Component

The first pathways involved in the structural development of the leaf tissue is the lignin biosynthetic pathway. Genes for lignin metabolism are enriched in immature tissue, along with those for hydroquinone: oxygen oxidoreductase activity, such as “laccases”, a class of enzymes known to be involved in the lignification of vascular tissue^42^. These lignification-related genes are upregulated as the vascular tissue in the leaf expands during development, and the lignified xylem provides structural reinforcement.

Immature leaf tissue also expresses an abundance of genes encoding enzymes that regulate pectin production and modification, such as aspartyl esterase and pectinesterase. The highly pectinaceous cell walls of the immature leaves are likely responsible for the great flexibility which protects growing and expanding leaves from structural damage^43^. The increase in pectin-digesting enzymes is expected in any tissue undergoing rapid cell division as cell wall polysaccharides are digested and modified during expansion and cytokinesis. Similar changes in pectin modification pathways have been well documented in the leaf venation of other species such as the potato (*Solanum tuberosum*) and mouse-ear cress (*Arabidopsis thaliana*)^44,45^.

Peroxidase enzymes used in cell wall modification are also up-regulated in immature leaf tissue. Hydrogen peroxide has been shown to play a signaling role in the modification and building of cell walls and other cell structures^46^. Expression of peroxidase genes was more abundant in immature leaf tissue, indicating their potential involvement in the development of these leaves.

### GO Enrichment in Mature Tissue

Table 2 lists selected GO terms related to housekeeping pathways, as well as oil and pigment production, upregulated in mature tissue, along with measures of their statistical significance. The majority of GO terms enriched in mature tissue were housekeeping genes, such as those involved in intracellular molecular transport (7 GO terms) and membrane production (3 GO terms). The upregulation of intracellular molecular transport genes could be a result of larger, fully differentiated cells that require more frequent and distant transport of intra- and extra-cellular materials than in immature tissue.

**Table 2:** Housekeeping, pigment production, and oil production GO terms significantly enriched in mature leaf tissue as a result of differential expression.

GO ID	*p*-value	# Genes Upregulated	GO Term	GO Category
GO:0008017	9.43E-05	5	microtubule binding	Molecular Function
GO:0007018	5.11E-04	4	microtubule-based movement	Biological Process
GO:0006869	1.04E-03	2	lipid transport	Biological Process
GO:0010333	2.75E-03	2	terpene synthase activity	Molecular Function
GO:0010430	3.44E-03	1	fatty acid omega-oxidation	Biological Process
GO:0046148	4.19E-03	1	pigment biosynthetic process	Biological Process
GO:0003777	5.54E-03	3	microtubule motor activity	Molecular Function
GO:0008574	1.21E-02	1	ATP-dependent microtubule motor activity	Molecular Function
GO:0008289	1.57E-02	2	lipid binding	Molecular Function
GO:0006839	1.57E-02	1	mitochondrial transport	Biological Process
GO:0005871	3.07E-02	1	kinesin complex	Cellular Component
GO:0000226	4.26E-02	1	microtubule cytoskeleton organization	Biological Process

The terpene synthase gene family (*p* = 0.0028) was upregulated in mature tissue, including one enzyme class that is possibly homologous to synthases that produce limonene, pinene, (E)-beta-ocimene, and (+)-alpha-phellandrene (sequence homology E-value = 0) terpenes. These have been shown to be constituents of the essential oil in the curry leaf and contribute significantly to the aroma and flavor of the plant^13–16,20,21^. Recently, several of the secondary metabolites produced by enzymes in this polyketide synthase gene family have been identified via cloning of transcripts in an expression vector and subsequent GC-MS chromatography. Many terpene synthase genes were also identified using transcriptomic sequencing in the curry leaf and were compared with those of other species in a phylogenetic context^31^. Unlike in the present study, however, differential expression of these genes was not studied during leaf development and oil gland morphogenesis. During our study, the expression of these terpene synthase family genes was compared during leaf development, and Fig. 9 shows a significant trend of upregulation of terpene synthase activity after leaf maturation. Another upregulated class of sesquiterpene synthases are those which produce caryophyllene (sequence homology E-value <10^−110^), a sesquiterpene oil that has been found to be the main constituent of curry essential oil^13,14,16,20^, yielding the various medicinal properties of the leaf^19^. This supports anatomical studies which show that oil gland counts continue to increase late into leaf development^47^. The upregulation of this class of enzymes indicates an increase in the production of these oils during leaf development. This corroborates with previous research that describes correlations between slower growing plants and an increased metabolic investment in antiherbivory compounds^48^. The upregulation in terpene synthases may also reflect an increase in gibberellin production. Fully mature leaf tissue may begin synthesizing gibberellic acid to regulate the onset of senescence and programmed cell death^25^.

The GO category for pigment biosynthesis was also enriched in mature leaf tissue (*p* = 0.0042), pigments which are necessary for the photosynthesis. The mature leaves have an increased cell count and size and a deeper green color than the nascent leaf tissue which appears etiolated (Fig. 1).

### Further study for differential expression validation

The findings reported here for differential expression between leaf growth stages are based solely on computational approaches to RNA-seq analysis. The transcriptome was assembled using read overlap from the original RNA-seq dataset, and gene expression was estimated using read alignment to the assembled transcriptome. Using reads aligned to a transcript as a proxy for the abundance of the transcript only provides a computational estimate, which, in order to make valid claims about differential expression, must be validated by various approaches. The sequence data is provided to assist in future studies aimed at validating these results, using methods such as quantitative polymerase chain reaction (qPCR), or repeating the study with multiple biological replicates. Thus, the results presented will aid in the further study of the gene expression profile of the curry species.

Another opportunity for future study is to investigate the change in the metabolic profile of the leaves through development, correlating the changes in gene expression of relevant enzymes involved in metabolic synthesis to the changes in metabolites. By analyzing the change in compounds such as terpenes and other products in the essential oil, the results of differential gene expression studies can be further validated.

## Conclusion

The transcriptome pattern of the curry leaf was a useful tool in the analysis of the differential expression of genes and pathways in immature and mature tissue. Until this study, only a few nuclear genes, complete chloroplast genome^49^, and a transcriptome^31^ in mature leaves had been sequenced for the curry tree; this study adds 57,383 newly sequenced transcripts (including multiple transcriptional isoforms of the same gene), contributing to a new understanding of the molecular genetics of the curry leaf development. The upregulated pathways identified in immature leaves can now be studied in this species, as well as offer molecular resources for studying the antiherbivory mechanisms of vulnerable meristematic tissues in the absence of mature oil glands. Lastly, the classes of enzymes involved in the synthesis of terpenes were characterized in curry leaves, and the differential expression of these terpene synthase genes was studied, with results showing a significant increase in terpene biosynthesis after leaf maturation. This will enable future studies to investigate the role of these enzymes and metabolites in the ecology and physiology of this species as well as its application in food and medicine.

## Data Availability

All sequence reads are available in the NCBI Sequence Read Archive (SRA) under the accessions SRR5590072 and SRR5590073.

## References

[1] *T**he Plant List* (2013). Version 1.1. Published on the Internet; http://www.theplantlist.org/ (accessed 30^th^ Sep, 2016).

[2] Morton CM, Telmer C (2014). New subfamily classification for the Rutaceae. Annals of the Missouri Botanical Garden.

[3] Mabberley DJ (1997). A classification of edibleC*itrus (*Rutaceae). Telopea..

[4] Iyer, D. & Devi, P. U. (2008). PHCOG REV.: Plant Review Phyto-pharmacology of *Murraya koenigii* (L.). *Pharmacognosy Rev*. **2** (1997).

[5] Kesari AN, Gupta RK, Watal G (2005). Hypoglycemic effects of *Murraya koenigii* on normal and alloxan-diabetic rabbits. J. Ethnopharmacology.

[6] Yadav S, Vats V, Dhunnoo Y, Grover JK (2002). Hypoglycemic and antihyperglycemic activity of *Murraya koenigii* leaves in diabetic rats. J. Ethnopharmacology.

[7] Vinuthan MK, Girish Kumar V, Ravindra JP, Narayana K (2004). Effect of extracts of *Murraya koenigii* leaves on the levels of blood glucose and plasma insulin in alloxan-induced diabetic rats. Indian J. Physiol. Pharmacol.

[8] Arulselvan P, Subramanian SP (2007). Beneficial effects of *Murraya koenigii* leaves on antioxidant defense system and ultra structural changes of pancreatic β-cells in experimental diabetes in rats. Chemico-Biological Interact..

[9] Tachibana Y, Kikuzaki H, Lajis NH, Nakatani N (2001). Antioxidative activity of carbazoles from *Murraya koenigii* leaves. J. Agric. Food Chem..

[10] Darvekar VM, Patil VR, Choudhari AB (2011). Anti-inflammatory activity of *Murraya koenigii* Spreng on experimental animals. J. Nat. Prod. Plant Resour..

[11] Nagappan T, Ramasamy P, Wahid MEA, Segaran TC, Vairappan CS (2011). Biological activity of carbazole alkaloids and essential oil of *Murraya koenigii* against antibiotic resistant microbes and cancer cell lines. Molecules.

[12] Ito C (2006). Induction of apoptosis by carbazole alkaloids isolated from *Murraya koenigii*. Phytomedicine.

[13] MacLeod AJ, Pieris NM (1982). Analysis of the volatile essential oils of *Murraya koenigii* and *Pandanus latifolius*. Phytochemistry.

[14] Wong KC, Tie DY (1993). The essential oil of the leaves of *Murraya koenigii* Spreng. J. Essent. Oil Res..

[15] Mallavarapu GR, Ramesh S, Syamasundar KV, Chandrasekhara RS (1999). Composition of Indian curry leaf oil. J. Essent. Oil Res..

[16] Chowdhury JU, Bhuiyan MNI, Yusuf M (2008). Chemical composition of the leaf essential oils of *Murraya koenigii* (L.) Spreng and *Murraya paniculata* (L.) Jack. Bangladesh J. Pharmacol..

[17] Jirovetz L, Buchbauer G, Ngassoum MB, Geissler M (2002). Aroma compound analysis of *Piper nigrum* and *Piper guineense* essential oils from Cameroon using solid-phase microextraction–gas chromatography, solid-phase microextraction–gas chromatography–mass spectrometry and olfactometry. J. Chromatography A.

[18] Guan W, Li S, Yan R, Tang S, Quan C (2007). Comparison of essential oils of clove buds extracted with supercritical carbon dioxide and other three traditional extraction methods. Food Chem..

[19] Ghelardini C, Galeotti N, Mannelli LDC, Mazzanti G, Bartolini A (2001). Local anaesthetic activity of β-caryophyllene. Il Farmaco.

[20] Verma RS, Padalia RC, Arya V, Chauhan A (2012). Aroma profiles of the curry leaf, *Murraya koenigii* L. Spreng. chemotypes: variability in north India during the year. Ind. Crops Prod..

[21] Deterre S (2012). Identification of key aroma compounds from bitter orange (*Citrus aurantium* L.) products: essential oil and macerate–distillate extract. Flavour and Fragrance J..

[22] Dagne E, Bisrat D, Alemayehu M, Worku T (2000). Essential oils of twelve *Eucalyptus* species from Ethiopia. J. Essent. Oil Res..

[23] Yu EJ, Kim TH, Kim KH, Lee HJ (2004). Aroma‐active compounds of *Pinus densiflora* (red pine) needles. Flavour and Fragrance J..

[24] Tholl D (2006). Terpene synthases and the regulation, diversity and biological roles of terpene metabolism. Current Opinion in Plant Biol..

[25] Smart CM (1994). Gene expression during leaf senescence. New Phytologist.

[26] Sesták, Z. (Ed.). *Photosynthesis During Leaf Development*. (Vol. 11). Springer Science & Business Media (2012).

[27] Chomczynski P, Sacchi N (1987). Single-step method of RNA isolation by acid guanidinium thiocyanate-phenol-chloroform extraction. Anal. Biochem..

[28] Ghawana S (2011). An RNA isolation system for plant tissues rich in secondary metabolites. BMC Research Notes.

[29] Grabherr MG (2011). Full-length transcriptome assembly from RNA-Seq data without a reference genome. Nature Biotechnol..

[30] Simão FA, Waterhouse RM, Ioannidis P, Kriventseva EV, Zdobnov EM (2015). BUSCO: assessing genome assembly and annotation completeness with single-copy orthologs. Bioinformatics.

[31] Meena, S. *et al*. Transcriptomic insight into terpenoid and carbazole alkaloid biosynthesis, and functional characterization of two terpene synthases in curry tree (*Murraya koenigii*). *Sci. Rep*. **7** (2017). 10.1038/srep44126 PMC5341033 28272514

[32] Altschul SF, Gish W, Miller W, Myers EW, Lipman DJ (1990). Basic local alignment search tool. J. Mol. Biol..

[33] Conesa A (2005). Blast2GO: a universal tool for annotation, visualization and analysis in functional genomics research. Bioinformatics.

[34] Götz S (2008). High-throughput functional annotation and data mining with the Blast2GO suite. Nucleic Acids Res..

[35] Ashburner M (2000). Gene Ontology: tool for the unification of biology. Nature Genet..

[36] Haas BJ (2013). De novo transcript sequence reconstruction from RNA-seq using the Trinity platform for reference generation and analysis. Nature Protoc..

[37] Li B, Dewey CN (2011). RSEM: accurate transcript quantification from RNA-Seq data with or without a reference genome. BMC Bioinformatics.

[38] Langmead B, Salzberg SL (2012). Fast gapped-read alignment with Bowtie 2. Nature Methods.

[39] Robinson MD, McCarthy DJ, Smyth G (2010). K. edgeR: a Bioconductor package for differential expression analysis of digital gene expression data. Bioinformatics.

[40] Gentleman RC (2004). Bioconductor: open software development for computational biology and bioinformatics. Genome Biol..

[41] Young MD, Wakefield MJ, Smyth GK, Oshlack A (2010). Gene ontology analysis for RNA-seq: accounting for selection bias. Genome Biol..

[42] O’Malley DM, Whetten R, Bao W, Chen CL, Sederoff RR (1993). The role of of laccase in lignification. The Plant J..

[43] Cosgrove DJ (2005). Growth of the plant cell wall. Nature Rev. Mol. Cell Biol..

[44] Pilling J, Willmitzer L, Bücking H, Fisahn J (2004). Inhibition of a ubiquitously expressed pectin methyl esterase in *Solanum tuberosum* L. affects plant growth, leaf growth polarity, and ion partitioning. Planta.

[45] Peaucelle A (2008). *Arabidopsis* phyllotaxis is controlled by the methyl-esterification status of cell-wall pectins. Current Biol..

[46] Potikha TS, Collins CC, Johnson DI, Delmer DP, Levine A (1999). The involvement of hydrogen peroxide in the differentiation of secondary walls in cotton fibers. Plant Physiol..

[47] Shanker S, Ajayakumar PV, Kumar S, Sangwan RS (1999). Essential oil gland number and ultrastructure during *Mentha arvensis* leaf ontogeny. Biol. Plant..

[48] Coley PD, Bryant JP, Chapin FS (1985). Resource availability and plant antiherbivore defense. Science.

[49] Shivakumar, V. S., Appelhans, M. S., Johnson, G., Carlsen, M. & Zimmer, E. A. Analysis of whole chloroplast genomes from the genera of the Clauseneae, the Curry tribe (Rutaceae, *Citru*s family). *Mol. Phylogenet. and**Evol*. **117**, 135–140 (2016). 10.1016/j.ympev.2016.12.015 27965082

